# Poorly differentiated synovial sarcoma of the vagina: a case report and a clinical literature review

**DOI:** 10.3332/ecancer.2008.99

**Published:** 2008-11-19

**Authors:** L Minig, G Farnetano, M Peiretti, G Roviglione, V Zanagnolo, G Pelosi, F Landoni

**Affiliations:** 1 Department of Gynecology, European Institute of Oncology, 20141 Milan, Italy; 2 Department of Pathology, European Institute of Oncology, 20141 Milan, Italy; 3 Department of Obstetrics and Gynecology, Casa di Cura Malzoni, Avellino, Italy

## Abstract

Synovial sarcomas (SS) account for 5–10% of soft-tissue sarcomas and typically arise in the para-articular regions of adolescents and young adults. Nonetheless, SS can occasionally occur in other regions of the body. Here, we present a first clinical literature report of a patient with an SS arising from the vaginal wall. A 40-year-old patient who presented a necrotic polypoid lesion, measuring 50 mm and extending from the external urethral meatus to the middle part of the anterior vaginal wall. The biopsy showed a poorly differentiated SS with abundant necrosis and a SYT-SSX1 mutation. A staging CT scan was negative for distant metastases. The patient, prior to the radical surgery, received neoadjuvant chemotherapy (ifosfamide and epirubicin) for three cycles. She underwent post-operative external radiotherapy and brachytherapy (50 Gy) due to close margins (<1 mm) in the pathologic specimen. She relapsed 11 and 16 months later with lung metastases, which, both times, were successfully removed by surgical resection. At 24 months from diagnosis, the patient is alive without further evidence of disease. In summary, in the presence of unfavourable prognostic factors, neoadjuvant chemotherapy could be the primary approach to reduce the tumour size and the risk of distant micro-metastases allowing a less aggressive radical surgery if the tumour is located in a non-extremity site. Hence, a multidisciplinary approach, if not influencing overall survival and disease-free survival, may improve the quality of life. In fact, in our patient we obtained a complete clinical control in the pelvis, avoiding pelvic exenteration with neoadjuvant chemotherapy.

## Background

Synovial sarcomas account for 5–10% of soft-tissue sarcomas, which typically arise in the para-articular regions of adolescents and young adults. Nonetheless, SS rarely occur in the mediastinum, head and neck region, heart, lung and retroperitoneum. Here, we present a clinical literature report of a patient with a synovial sarcoma arising from the vaginal wall. The immunohistochemical, molecular and ultrastructural details of this case have been previously reported [1].

## Case presentation

A 40-year-old patient was referred to the European Institute of Oncology (Milan, Italy) with a diagnosis, from elsewhere, of a high-grade vaginal leiomyosarcoma. She was severely anaemic due to abnormal vaginal bleeding, and pelvic examination revealed a necrotic, friable and bluish polypoid lesion, measuring 50 mm longitudinally and extending from the external urethral meatus to the middle part of the anterior vaginal wall. A staging CT scan was negative for distant metastases.

Histopathological review of the original sections revealed a poorly differentiated synovial sarcoma, and the patient was treated with neoadjuvant chemotherapy, including high-dose ifosfamide (9 g/m^2^) and epirubicin (90 mg/m^2^), q 21 days, for three cycles, obtaining a partial clinical response (tumour size by clinical examination: 1 cm). She then underwent wide local excision of the anterior vaginal wall, with preservation of the external urethral meatus.

The pathology report confirmed the diagnosis of a poorly differentiated synovial sarcoma and the patient received post-operative external radiotherapy and brachytherapy (50 Gy) for close margins (<1 mm).

Eleven months later the patient experienced three metastases to the right lung, which were treated with wedge resection. Five months later, three other metastases were found on the contralateral lung and were successfully removed by surgical resection.

At 25 months from diagnosis, the patient is alive without further evidence of pulmonary or other metastases or local recurrence.

### Pathological and molecular findings

Microscopic examination of this case revealed a high-grade, biphasic, poorly differentiated synovial sarcoma with abundant necrosis and mitoses (more than 10/10 high-power fields). The immunohistochemical findings were consistent with the diagnosis of a poorly differentiated synovial sarcoma, arising from the vaginal wall.

The rearrangement of the SYT gene, characteristic of SS, was detected confirming the diagnosis of synovial sarcoma. A reverse transcriptase-polymerase chain reaction assay revealed a SYT-SSX1 fusion gene product of 158 bp.

## Discussion

Synovial sarcomas constitute approximately 5–10% of all soft-tissue sarcomas [2]. Although 90% of cases are diagnosed in the extremities and occur before the age of 50 years, they may arise in a wide variety of organs, including head and neck area, mediastinum, heart, lung, abdominal wall, mesentery, retroperitoneum and peritoneal cavity [3]. In the female genital tract, the occurrence of synovial sarcomas is extremely rare, and only isolated case reports have been described in the fallopian tube, [4] the vulva [5–7] and the vagina [8].

The chance of a synovial sarcoma occurring in other body locations apart from the extremities is supported by the hypothesis that they do not arise from synovial tissue. In fact, it has been suggested that they originate from unknown stem cells that are capable of differentiating into mesenchymal and/or epithelial structures (an hypothesis that still needs to be confirmed) [9]. Biphasic synovial sarcomas contain both epithelial cells arranged in glandular structures and spindle cells, whereas monophasic types are entirely composed of spindle cells. On the other hand, the poorly differentiated type, as the one reported here, often contain small round cells resembling those seen in Ewing's primitive neuroectodermal tumours. However, only immunohistological, ultrastructural and cytogenetic features allow correct diagnosis.

Other types of synovial sarcoma include leiomyosarcoma (which account for the most common histology in any single tumour series of the vagina), the spindle cell variant of squamous cell carcinoma and the rare mixed tumour of the vagina, either benign or malignant.

Synovial sarcomas are characterized by the presence a specific genetic translocation, the SYT-SSX translocation t(X;18) (p11.2;q11.2), which represents a specific diagnostic marker, being present in 95% of all SS cases. Its presence, as the only cytogenetic abnormality, suggests that it is the primary cause of synovial sarcoma.

There are several subtypes, numbered SYT-SSX 1, 2 and 4 [10,11], and these genetic subtypes are correlated with histological subtypes and clinical behaviour. Biphasic synovial sarcomas are associated with the presence of the SYT-SSX1 mutation, while monophasic synovial sarcomas are correlated with the mutation SYT-SSX2. There are data that indicate that a particular subtype of translocation has prognostic significance. In two studies, the SYT-SSX 2 translocation was primarily associated with the monophasic histotype and a better outlook for survival five years after diagnosis, compared with the SYT-SSX 1 translocation, which was primarily associated with biphasic disease and a worse outlook for five-year survival [10,11].

An essential first step in the successful treatment of synovial sarcoma is adequate surgical resection with tumour-free surgical margins [12–14].

However, in cases with unfavourable localization such as the head and neck, retroperitoneum, chest and female genital tract, chemotherapy and, perhaps, pre-operative radiation therapy should be initially given, with the aim of reducing the tumour volume, thus facilitating surgical resection. Our patient received primary chemotherapy with epirubicin and ifosfamide, with good partial remission. Other active regimens can include vincristine, actinomycin D and cyclophosphamide (VAC).

It is interesting to point out that the combination of doxorubicin and ifosfamide induced a more significant responses compared to VAC regimens, as described in two other studies [15,16].

After surgery, our patient received 50 Gy of radiotherapy, as the tumour margins were close (<1 mm) in the pathologic specimen. Given sarcoma's relative rarity, the precise doses of radiotherapy to achieve local control in microscopic and gross residual disease categories are not well known. However, Raney *et al* [17] usually propose to use a dose of 55 Gy for microscopic or recurrent disease after surgical removal, and somewhat higher, about 60 Gy, in the presence of visible residual tumour.

The natural course of the disease is such that many patients will experience recurrence of the primary tumour and/or metastatic disease. The reported five-year overall survival rates range from 38% to 76% [18,19] and from 20% to 63% in ten years [19,20]. As synovial sarcomas can grow slowly, the ten or 20-year survival rates are more accurate. In fact Paulino [21] found that approximately 26% of distant relapses occurred after five years from the initial diagnosis and 2% were diagnosed 15 years after the initial diagnosis. Metastatic disease occurs in almost 50% of patients. The most common site of metastasis is the lungs (74–81%), lymph nodes (3–23%) and bone (10–20%) [22–24].

The unfavourable prognostic factors, described in several studies, include a tumour size more than 5 cm, tumour localization in a non-extremity site, primary treatment outside a referral centre, a patient age of more than 25 years, the presence of tumour necrosis areas and bone invasion [13,25] and the presence of a particular SYT-SSX translocation (SYT-SSX1) [10]. However, the strongest prognostic factor associated with local recurrence, metastases and tumour-related death in a multivariate analysis [25] is the presence and the amount of poorly differentiated areas within the tumour.

Our patient was more than 25 years old, had a tumour of more than 5 cm with abundant necrosis and poorly differentiated histology, a non-extremity site location and an SYT-SSX1 mutation. All these factors indicate an unfavourable prognostic outcome and can probably explain the aggressive behaviour of the tumour.

In summary, in the presence of unfavourable prognostic factors, neoadjuvant chemotherapy could be the primary approach to reduce the tumour size and the risk of distant micro-metastases and allowing a less aggressive radical surgery if the tumour is located in a non-extremity site. Hence, a multidisciplinary approach, even if having no influence on overall survival and disease-free survival, may improve the quality of life. In fact, in our patient we obtained complete clinical control in the pelvis with neoadjuvant chemotherapy, so avoiding pelvic exenteration.
